# Ultrasonography-Guided Dextrose Prolotherapy Versus Platelet-Rich Plasma Injections for the Treatment of Plantar Fasciitis: A Randomized Controlled Trial

**DOI:** 10.7759/cureus.77566

**Published:** 2025-01-16

**Authors:** Neelam Kumari, Sudhir R Mishra, Anil K Gupta, Tulika Chandra, Dileep Kumar, Ganesh Yadav, Sunil Kumar

**Affiliations:** 1 Department of Physical Medicine and Rehabilitation, King George's Medical University, Lucknow, IND; 2 Department of Transfusion Medicine, King George's Medical University, Lucknow, IND

**Keywords:** dextrose prolotherapy, pain intensity scale, plantar fasciitis, platelet-rich plasma (prp), ultrasound-guided, usg-guided dextrose prolotherapy

## Abstract

Introduction: Heel pain is prevalent in foot and ankle practice, with plantar fasciitis being a common cause of chronic heel pain in adults. It is a degenerative condition of the plantar fascia, rendering the term fasciitis inaccurate. The condition has several alternative names, including painful heel syndrome and heel spur syndrome. It is found that a significant number of people suffer from plantar fasciitis at some stage of their lives; athletes and women between 40 and 60 years and adults aged more than 65 suffer the most. Patients typically report tenderness at the medial aspect of the heel, and pain is mostly under the surface of the heel, especially with initial morning steps. Plantar fasciitis is predominantly unilateral. Treatment options include prolotherapy and platelet-rich plasma (PRP), with this randomized controlled trial (RCT) aiming to evaluate the efficacy of PRP versus 25% dextrose prolotherapy in alleviating plantar fasciitis symptoms.

Aims and objectives: This study aimed to assess the comparative effectiveness of USG-guided 25% dextrose prolotherapy and autologous PRP injections on pain intensity, foot function, and ankle outcomes in plantar fasciitis cases.

Methodology: The study took place at the Department of Physical Medicine and Rehabilitation (PMR), King George’s Medical University (KGMU), Lucknow, Uttar Pradesh, India, with eligible patients evaluated. Group A received 25% dextrose prolotherapy, while Group B was administered autologous platelet-rich plasma. Outcome measures, including pain by Pain Intensity Scale (PIS) and assessment of disability by Foot Function Index (FFI) and Foot and Ankle Outcome Score (FAOS), were assessed at baseline and the third and sixth weeks after intervention.

Results: A total of 44 patients were randomized into two groups of 22. Group A received dextrose prolotherapy, while Group B was treated with PRP injections as per the study protocol. The average age of participants in our study was 36.10±10.0 years. Female participants were more affected than male participants in both groups. Overall, the male-female ratio was 38.6:61.4, while in Group A and Group B, this ratio was 45.5:54.5 and 31.8:68.2, respectively. The presentation of plantar fasciitis was bilateral (40.9%), on the right side (40.9%), and on the left side (18.2%). The right side was more frequently involved than the left side of the plantar fascia. There was a decrease in the average PIS score of Groups A and B at the baseline, which was 7.27±1.24 and 7.55±1.26, respectively, which was reduced to 3.18±1.30 and 2.50±0.67 after six weeks of follow-up. The average FFI score of Groups A and B at the baseline was 98.64±17.68 and 95.36±19.01, respectively, which was reduced to 79.77±12.77 and 63.77±18.50 after six weeks of follow-up. The average FAOS score of Groups A and B at the baseline was 71.36±13.95 and 74.86±28.72, respectively, which was reduced to 74.86±28.72 and 42.09±5.03 after six weeks of follow-up.

Conclusion: Both 25% dextrose prolotherapy and PRP injections are effective treatments for plantar fasciitis. Both are minimally invasive and safe treatments for plantar fasciitis.

## Introduction

Heel discomfort is a prevalent presenting grievance in the podiatric and ankle domain, and plantar fasciitis represents the foremost common etiology of persistent discomfort beneath the heel in adults, constituting 11%-15% of the podiatric symptoms necessitating professional intervention among adults [1]. It is a degenerative ailment of the plantar fascia insertion; hence, the designation fasciitis is a misnomer. In the extant literature, it has been characterized as painful heel syndrome, chronic plantar heel discomfort, heel spur syndrome, runner's heel, and calcaneal periostitis [2]. It is projected that one in 10 individuals will encounter plantar fasciitis at some stage throughout their lifespan [3]. Females aged 40-60 years are the most frequently impacted [4]. Although the incidence of plantar fasciitis is more prevalent in the athletic population, ranging from 4.5% to 10%, it also adversely affects the sedentary demographics, with 7% of adults aged over 65 years [5-6]. The inherent trajectory of plantar fasciitis is often self-resolving. Nevertheless, the average time frame for resolution typically ranges from six to 18 months, although it may be considerably prolonged if the condition remains undiagnosed [7].

With prompt diagnosis and timely implementation of nonsurgical interventions such as activity modification, gastrocnemius and plantar fascia-specific stretching, anti-inflammatory pharmacotherapy, and/or orthotic shoe inserts, the prognosis is favorable, with approximately 80% of patients attaining symptom resolution within one year [8]. The plantar fascia, or deep fascia of the sole, possesses a direct fibrocartilaginous attachment to the calcaneum proximally. It exhibits a triangular morphology and originates from the medial process of the calcaneal tuberosity. The calcaneum is demarcated from the plantar dermis by a complete honeycombed fibro-fatty adipose pad that functions as a shock absorber [9]. The fascia is multilayered and exhibits a thickness of approximately 3 mm [10]. The patient typically articulates discomfort along the medial aspect of the foot at the inferior aspect of the heel. This is most pronounced with initial ambulation after a period of inactivity, and the painful first steps upon awakening in the early morning generally diminish with an increased level of activity throughout the day, although it tends to exacerbate towards the day's conclusion [11]. The diagnostic approach for plantar fasciitis is the windlass test, which possesses a specificity of 100% and a sensitivity of 32%. The test is deemed positive if it reproduces or exacerbates the patient's discomfort. The windlass mechanism transpires during the terminal stance phase when the heel is elevated off the ground. Plantar fasciitis is typically unilateral, yet up to 40% exhibit a bilateral manifestation and tightness of the Achilles tendon is observed in nearly 80% of cases [12-13].

For the management of plantar fasciitis, interventions such as prolotherapy have been employed, whereby a small volume of irritant solution (proliferant) is injected at multiple locales surrounding the ligament or tendon insertion. This solution elicits a localized inflammatory response at the injection site, which stimulates fibroblast proliferation and subsequent collagen synthesis due to an ensuing upregulation and migration of various growth factors responsible for tissue repair [14]. Platelet-rich plasma (PRP) constitutes an alternative modality for the management of plantar fasciitis, wherein an autologous product is extracted from whole blood via the mechanism of gradient density centrifugation. Platelet-rich plasma is characterized as blood possessing concentrations of platelets that exceed baseline levels, which are derived from the patient’s own blood [15-17]. Platelets encompass numerous critical bioactive proteins and a minimum of seven growth factors that may facilitate the repair of cartilage defects and promote the healing of the plantar fascia [18]. This randomized controlled trial (RCT) design is purposed to evaluate the efficacy of PRP therapy in comparison with 25% dextrose prolotherapy in alleviating pain intensity and symptoms associated with plantar fasciitis.

## Materials and methods

This study was conducted in the Department of Physical Medicine and Rehabilitation (PMR), King George’s Medical University (KGMU), Lucknow, Uttar Pradesh, India. After obtaining approval of ethical clearance from the Institutional Ethical Committee with reference code ECR/262/Inst/UP/2013/RR-19, dated 30/05/2020, it was also registered in the Clinical Trials Registry of India with registration number CTRI/2021/08/035572. All the patients satisfying the inclusion and exclusion criteria of the study (described below) were enrolled in the outpatient department (OPD) and inpatient department (IPD) from different departments. The recruitment methods used in this study were according to the Consolidated Standards of Reporting Trials (CONSORT) RCT reporting guidelines.

This is a single-blinded RCT. The duration of the study was between January 2020 and June 2021.

The sample size was calculated on the basis of variation in Foot Function Index (FFI) change in study groups using the following formula: 

n = (Zα+Zβ)2 (σ12 + σ22)/d2

where σ1 = 82.5. The half range of FFI change in the study group was σ2 = 31.52. The half range of FFI changes under the null hypothesis as found in the control group [19-20]; d = mean (σ1, σ2), the minimum mean difference was considered to be clinically significant, type I error α = 5% corresponding to a 95% confidence level, type II error β = 20% for detecting results with 80% power of study, data loss = 10%. So the required sample size came out to be n = 28 for each group. Eligible subjects were randomized using a computer-generated table with a 1:1 allocation ratio. The study aimed to enroll at least 56 subjects (28 per group), but only 44 subjects (22 per group) were enrolled due to the COVID-19 pandemic.

Inclusion criteria included participants aged between 18-60 years with a diagnosis of idiopathic plantar fasciitis. Patients with unresponsive plantar fasciitis after two weeks of conservative treatment and those who consented to participate in the study were included. Exclusion criteria included participants refusing to engage in the study, who had acute ankle or foot trauma, diagnosis of calcaneal fracture or stress fracture, the occurrence of acute infections such as cellulitis or septic arthritis, the existence of an open heel wound, diagnosis of calcaneal osteomyelitis, presence of entrapment neuropathy, and a history of plantar fascia surgery. The exclusion criteria also applied to those receiving local steroid injections within one month, using non-steroidal anti-inflammatory drugs or acetylsalicylate within 72 hours, participants with hemoglobin levels below 10 gm/dl, platelet counts under 150,000 per microliter of blood, individuals with uncontrolled diabetes mellitus, and those with a diagnosis of malignancy. 

Preparation of autologous PRP 

After obtaining informed consent, the patient was taken to the Department of Transfusion Medicine of KGMU. A standard double centrifugation protocol was used for PRP preparation, which includes the following steps: 20 ml of blood was withdrawn from the patient under aseptic conditions from an autologous donor in a quadruple bag that contained the anticoagulant citrate phosphate dextrose adenine (CPDA-1). The blood was processed in the component lab. The sedimentation of PRP was done by differential procedure. All the procedures were done in a closed system. The quadruple bag was placed in a centrifugation machine for eight minutes and centrifuged at a speed of 890 rpm (soft spin). After that, supernatant plasma containing platelets was transferred into a platelet bag without anticoagulant and then again centrifuged, but at a speed of 3150 rpm (hard spin) for 11 minutes to obtain a platelet concentrate. The upper 2/3^rds^, which was platelet-poor plasma (PPP), was discarded, and the lower 1/3^rd^ of PRP (approximately 5 ml) was obtained in a closed bag. The resulting concentration was three to four times more concentrated with platelets as compared to whole blood. The collected PRP bag was taken to the PMR department's operation theater (OT). Under aseptic precautions, 2 mL of PRP was withdrawn from the bag and injected into the origin of the plantar fascia under USG guidance in an out-of-plane approach within 30 minutes, using a 21-gauge needle. The patient was then looked at for any complications or side effects for 15 minutes, and after that, discharged.

All the patients satisfying inclusion and exclusion criteria were evaluated from baseline Pain Intensity Scale (PIS), FFI, and Foot and Ankle Outcome Score (FAOS) and grouped. Group A received 25% dextrose prolotherapy (1 ml of 25% dextrose plus 1 ml of 2% lignocaine). Group B received 2 ml of autologous PRP. Both received their respective injections under USG guidance with all aseptic precautions. All subjects were telephoned within 48 hours after injection to ensure that no adverse events occurred. All the patients were followed up in the third and sixth weeks for evaluation of the PIS, FFI, and FAOS. Assessment of pain was done by the PIS at the baseline, third, and sixth week after injection, and assessment of foot disability was done by the FFI and FAOS at the baseline, third, and sixth week after the injection.

Data entry was made in Microsoft Office Excel (Microsoft Corp., Redmond, WA) in codes, and analysis was done by IBM SPSS Statistics software, version 23 (IBM Corp., Armonk, NY). Descriptive statistical analysis, which included frequency, percentages, mean, and standard deviation, was used to characterize the data. Association with the factors was tested for significance using the chi-square test, and p < 0.05 was considered statistically significant. The intergroup comparisons were made by unpaired t-test.

## Results

In this study, we found that female participants were more affected than male participants in both groups. Overall, the male-female ratio was 38.6:61.4, while in Group A and Group B, this ratio was 45.5:54.5 and 31.8:68.2, respectively. The presentation of plantar fasciitis was bilateral (40.9%), on the right side (40.9%), and on the left side (18.2%). On assessing the laterality, the right side was more frequently involved than the left side of plantar fasciitis. The mean age of Groups A and B was 35.05±8.38 and 36.77±10.0 years, respectively. No significant difference was found in mean ages between the groups (p-value = 0.538). The mean height of Groups A and B was 157.91±11.43 cm and 160.09±7.95 cm, respectively. No significant difference was found in mean heights between the groups (p-value = 0.466). The mean weight of Groups A and B was 68.05±12.82 kg and 67.91±7.51 kg, respectively. No significant difference was found in mean weights between the groups (p-value = 0.966). The mean BMI of Groups A and B was 27.19±3.52 kg/m² and 26.57±2.92 kg/m², respectively. No significant difference was found in mean BMI between the groups (p-value = 0.529).

In Group A, the mean baseline PIS, FFI, and FAOS were 7.55, 98.64, and 74.86, respectively, and in Group B, the mean baseline PIS, FFI, and FAOS were 7.27, 95.36, and 71.36, respectively.

**Figure 1:**
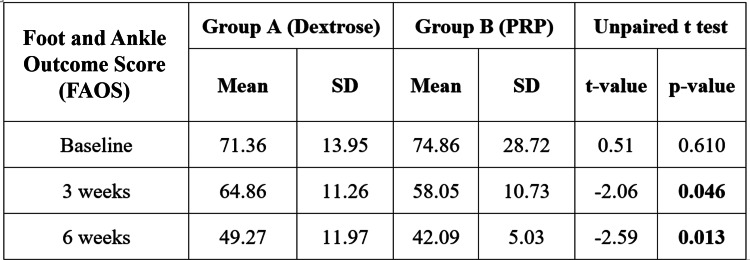
Intergroup comparison of Foot and Ankle Outcome Score (FAOS) The mean FAOS score of Group A was 71.36±13.95 at baseline, which was reduced to 49.27±11.97 after six weeks. In Group B, the mean FAOS score at baseline was 74.86±28.72, which was reduced to 42.09±5.03 after six weeks of follow-up. PRP: platelet-rich plasma

**Figure 2:**
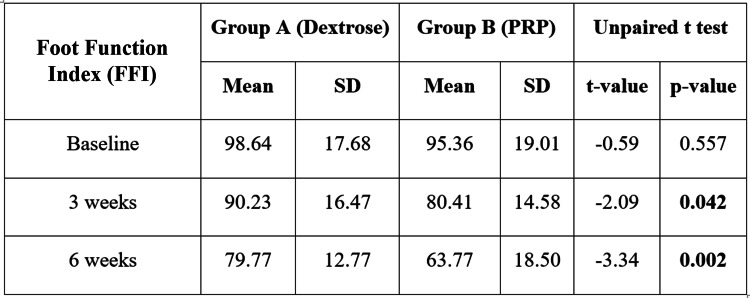
Intergroup comparison of the Foot Function Index (FFI) The mean FFI score of Group A was 98.64±17.68 at baseline, which was reduced to 79.77±12.77 after six weeks. In Group B, the mean FFI at baseline was 95.36±19.01, which was reduced to 63.77±18.50 after six weeks of follow-up. PRP: platelet-rich plasma

**Figure 3:**
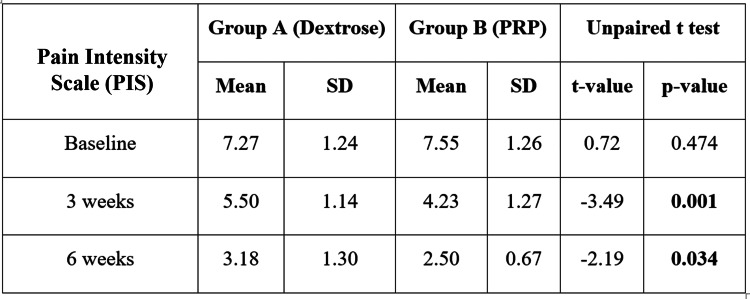
Intergroup comparison of the Pain Intensity Scale (PIS) The mean PIS score of Groups A and B at the baseline was 7.27±1.24 and 7.55±1.26, respectively, which was reduced to 3.18±1.30 and 2.50±0.67, respectively, after six weeks of follow-up. PRP: platelet-rich plasma

We also found the mean FAOS score of Group A was 71.36±13.95 at baseline, which was reduced to 49.27±11.97 after six weeks. In Group B, the mean foot and ankle outcome score at baseline was 74.86±28.72, which was reduced to 42.09±5.03 after six weeks (Figure 1). No significant difference was found in the mean FAOS at baseline (p-value = 0.610); afterward, the difference was significant in the third week (p-value = 0.046) and sixth week (p-value = 0.013). After six weeks, more reduction was observed in Group B. The mean FFI score of Group A was 98.64±17.68 at baseline, which was reduced to 79.77±12.77 after six weeks. In Group B, the mean FFI at baseline was 95.36±19.01, which was reduced to 63.77±18.50 (Figure 2). No significant difference was found in the mean FFI at baseline (p-value = 0.557); afterward, the difference was significant in the third week (p-value = 0.042) and sixth week (p-value = 0.002). After the sixth week, more reduction in FFI score was observed in Group B. The mean PIS score of Groups A and B at the baseline was 7.27±1.24 and 7.55±1.26, respectively, which was reduced to 3.18±1.30 and 2.50±0.67, respectively, after the six-week follow-up (Figure 3). No significant difference was found in the mean pain score at baseline (p-value = 0.474), but a significant difference was found at the third week's follow-up (p-value = 0.001) and the sixth week's follow-up (p-value = 0.034). On comparing both groups, the outcomes of Group B were found superior to Group A.

## Discussion

The average age of participants in our study was 36.10±10.0 years, which is similar to the 37.8 years reported by Kim and Lee [20] and the 39.0±8.8 years noted by Kalia et al. [21]. In contrast, Wrobel et al. [22] reported a mean age of 49.7±12.4 years, as their study included patients of all ages with plantar fasciitis, while ours focused on individuals aged 18 to 60 years. Ragab and Othman [23] recorded an 88% reduction in pain after PRP injections, which is consistent with our results of significant improvement at six weeks, although significance was evident at 12 months. Tiwari et al. [24] demonstrated significant improvement in the PRP group compared to local steroid injections, while our study showed significant results at six weeks compared to dextrose and steroid groups. Kim and Lee [20] found that the average pain scores on the baseline scale were 56.5±14.0 for the dextrose prolotherapy group and 60.4±14.7 for the PRP group, showing a clear improvement in the PRP group (p<0.001). Our research showed a decrease in the average PIS score from 7.27±1.24 at the start to 3.18±1.30 at the sixth-week follow-up, supported by Ersen et al. [25]. Deghady et al. [19] noted a reduction in the Visual Analog Scale (VAS) scores following PRP injections, matching our study's follow-up periods of the third and sixth weeks. Kalia et al. [21] observed significant improvements in VAS scores after a single PRP injection, similar to our findings in the sixth and 12th weeks (p < 0.001). Siddiqui et al. [26] reported notable improvements in VAS scores following PRP treatment, which aligns with the significant PIS score improvements seen in our study in the sixth week.

Demir et al. [9] discovered significant improvements in FFI scores among patients treated with dextrose prolotherapy for plantar fasciitis, consistent with our observation of greater improvement in group B compared to group A. The study by Kim and Lee [20] indicated that the mean FFI scores decreased significantly in the PRP group compared to the DP group, with notable changes observed earlier in our study. Our research revealed that a single injection of DP led to a decrease in mean FFI scores, in line with Ersen et al. [25], who reported significant improvements after multiple dextrose prolotherapy injections. After giving a single injection of PRP, our study showed a substantial decrease in mean FFI scores, in agreement with the results of Deghady et al. [19]. We also observed a significant improvement in FAOS scores after PRP injection, supporting the findings of Kumar et al. [27], who noted significant improvements following a similar treatment. Our study reported better FAOS scores after dextrose prolotherapy, mirroring the significant outcomes seen in Ersen et al. [25] following multiple dextrose prolotherapy sessions. Kalia et al. [21] found significant increases in FOAS scores after PRP treatment, which closely resembled our results. Siddiqui et al. [26] reported marked improvements in FAOS scores after PRP administration, while our study also noted significant enhancements in FAOS scores at the sixth-week follow-up.

Traditional healing methods include physical therapy, custom shoes, corticosteroid injections, extracorporeal shock wave therapy (ESWT), and surgery as a last resort. There is no universally accepted gold standard for treatment. Pain can linger for months, resulting in significant disability. This study found that 25% dextrose prolotherapy (Group A) and PRP injections (Group B) effectively reduced symptoms of plantar fasciitis, as indicated by improvements in the PIS, FFI, and FAOS. Although PRP showed better overall effectiveness than dextrose prolotherapy, no statistical significance was found in the PIS, FFI, and FAOS during follow-up.

Limitations

The limited number of participants was due to challenges in recruiting patients within the time frame and the impact of the COVID-19 pandemic. This study was carried out as a single-blinded randomized controlled trial, which might have affected the outcome. Additionally, the short follow-up duration in the study also impacted the results. Future research should focus on increasing the sample size, extending the duration of follow-ups, and implementing double-blinded methodologies.

## Conclusions

Both PRP injections and 25% dextrose prolotherapy have shown effectiveness in treating plantar fasciitis, with either method being a suitable option. Platelet-rich plasma therapy is suggested over 25% dextrose prolotherapy in situations where there are no contraindications. They both enhance pain relief and foot function and reduce disability in the short term. These methods are gentle, safe methods for addressing plantar fasciitis. However, PRP preparation is a more costly and complicated process as compared to 25% dextrose prolotherapy. Either procedure is suitable for patients with unsuccessful conservative management. Platelet-rich plasma therapy appears superior to 25% dextrose prolotherapy injection, though this difference is statistically non-significant. Further long-term studies with larger sample sizes are necessary to ascertain the superiority of single injections of 25% dextrose prolotherapy versus PRP.
